# Self-Regulated Resource Management in Emergency Remote Higher Education: Status Quo and Predictors

**DOI:** 10.3389/fpsyg.2021.672741

**Published:** 2021-06-02

**Authors:** Nick Naujoks, Svenja Bedenlier, Michaela Gläser-Zikuda, Rudolf Kammerl, Bärbel Kopp, Albert Ziegler, Marion Händel

**Affiliations:** ^1^Department of Education, Friedrich-Alexander University Erlangen-Nuremberg, Erlangen, Germany; ^2^Department of Psychology, Friedrich-Alexander University Erlangen-Nuremberg, Erlangen-Nürnberg, Germany

**Keywords:** emergency remote teaching, resource management strategies, digital readiness, higher education, self-regulated learning

## Abstract

Because of the COVID-19 pandemic in the spring term 2020, students faced a sudden change from on-campus learning to online learning with synchronous and asynchronous online courses (emergency remote teaching). To study successfully, students not only needed to be prepared in terms of digital readiness (workspace, IT equipment, previous online learning experiences, and sharing information online), they also faced challenges that pertained to the self-regulated management of external resources (environment structuring, time management, and help-seeking). In the current study, we investigated students’ digital readiness for the sudden switch to online learning; differences between students’ intended and actual use of external resource management strategies; and the influence of students’ digital readiness on their actual use of resource management strategies. Students enrolled in a full-scale, German university (*N* = 662) answered two online questionnaires (before and in the middle of the term). Descriptive statistics indicated that students seemed to be ready to study online. However, repeated measures ANOVA showed that students were not able to manage their resources during the term as frequently as intended. Finally, separate regression analyses revealed that availability of workspace and IT equipment predicted the use of environment structuring strategies. Additionally, IT equipment and information sharing behavior predicted students’ help-seeking. Based on the current results, we discuss implications for the promotion of student self-regulated learning (SRL) in online emergency remote teaching based on both external resources and digital readiness.

## Introduction

Learning in higher education institutions requires students to manage their learning process, that is, to self-regulate their learning (Dresel et al., 2015). More specifically, online and distance learning settings with high demands on student autonomy require skills to self-regulate one’s learning (Barnard et al., 2009; Bol and Garner, 2011). This applies especially to the spring term 2020, the first online term to deliver all learning material remotely. Due to the COVID-19 pandemic, on-site universities had to switch immediately to online distance education. This so-called emergency remote teaching (Hodges et al., 2020) produced several residual effects. Students’ learning material changed from predominantly paper-and-pencil to digital tools and materials. Their learning spaces changed from classrooms or libraries to their homes, and regular in-person higher education courses were either asynchronous or synchronous online courses. These changes placed tremendous demands on students’ self-regulated learning (SRL), especially for student application of strategies that regulate their resources.

For example, more so during the COVID-19 pandemic than under regular circumstances, students needed to regulate their physical learning environment. That is, students needed to find a suitable place to study while avoiding possible distractions during at-home study periods. Additionally, students had to keep track of their time management because of the partial absence of regular weekly courses, the mix of synchronous and asynchronous courses, and self-paced processing of asynchronous learning materials. Finally, they had to find new ways to communicate with their peers and lecturers, especially when they were seeking help. For example, it might have been more difficult to start an informal conversation with lecturers or fellow students due to the intermingling of asynchronous and synchronous events.

In the current study, we investigated how students coped with these changing and challenging learning conditions. First, we analyzed students’ preconditions (i.e., equipment, skills) to study exclusively online. Second, we investigated students’ use of resource management strategies with a focus on structuring the learning environment, time management, and help-seeking. Finally, we analyzed how students’ use of resource management strategies was related to their intended use of those strategies, as well as students’ preconditions for learning remotely.

### Theoretical Background

#### Demands of Online Learning

Online learning can be distinguished by several characteristics like modality (fully online, blended, and web-enabled face-to-face), pacing (self-paced or class-paced), assumed student roles, or synchronicity (Moore et al., 2011; Means et al., 2014). However, online learning at the beginning of the COVID-19 pandemic cannot be transferred 1:1 to these types of online learning scenarios as students faced a mix of different types of e-learning schemes. Teachers and students did not voluntarily decide to participate in online learning, but the unique circumstances brought about by the COVID-19 pandemic forced them to do so (Means and Neisler, 2020). Accordingly, what transpired during the spring term 2020 can be considered as a new type of online learning, labeled emergency remote teaching, or emergency remote education (Bozkurt et al., 2020; Hodges et al., 2020).

Students had nary any time to prepare for this exceptional situation and as a result, may have embarked on the online learning term with different preconditions (Beaunoyer et al., 2020; Czerniewicz et al., 2020; Händel et al., 2020a). In order to optimally participate in online education, students needed a quiet workspace and appropriate IT equipment, such as computer hard- and software (e.g., notebook and internet access). In addition to technical equipment, students had to depend on computer literacy skills and had to ask for information regarding course content and organizational aspects to cope with the new mode of learning. Hong and Kim (2018) refer to such actions of students as information sharing behavior. In line with Hong and Kim (2018), the European Council (2006) argues that students should be able to use “computers to retrieve, assess, store, produce, present, and exchange information and to communicate [online]” (p. 13). Indeed, to meet educational aims, students needed abilities to participate in courses that exclusively relied on web-based material and web-based interaction (Hong and Kim, 2018; Küsel et al., 2020). For the spring term 2020 in particular, students had to meet these conditions to ensure successful participation in emergency remote teaching. Therefore, and in contrast to regular (on-site) terms, students’ digital readiness to participate in emergency remote teaching is displayed by their workspace availability, equipment, previous experiences with online learning, and information sharing behavior.

In general, online learning environments are more self-paced than on-site and in-person learning situations (McBrien et al., 2009; Broadbent, 2017; Bruso et al., 2020). Students have higher autonomy regarding place and time, where and when to study. Accordingly, self-regulation becomes a critical factor for success in online learning (Jansen et al., 2017; Kocdar et al., 2018); for a comprehensive overview, we refer to recent review articles (Hodges, 2005; Broadbent and Poon, 2015; Garcia et al., 2018; Wong et al., 2019; Anthonysamy et al., 2020a; Martin et al., 2020). For example, in a study with students from blended learning courses, students experienced greater possibilities to self-regulate their learning in online learning situations than they did for in-person learning conditions (Lee and Tsai, 2011). However, students with more experience with online courses did not necessarily make more use of online self-regulated learning strategies (Bruso et al., 2020). In essence, emergency remote teaching may force students to face an even greater need to self-regulate their learning resources compared to students who chose to participate in self-paced, distance learning environments (Carter et al., 2020).

#### Resource Management Strategies in Online Learning Environments

Self-regulated learning means that students plan, monitor, and regulate their learning (Winne and Hadwin, 1998; Panadero, 2017). Models of self-regulated learning usually distinguish three main types of learning strategies – namely cognitive, metacognitive, and resource management strategies (Pintrich et al., 1991; Pintrich, 1999). While cognitive and metacognitive strategies are concerned ways of learning to understand content (e.g., *via* elaborating on the content or *via* monitoring understanding), resource management strategies pertain to the design of individual learning conditions. The current research focused on resource management strategies toward creating optimal learning conditions (Waldeyer et al., 2019). We argue that under the conditions of a pandemic with physical distancing, restricted access to campus or libraries, and changing formats of learning (shift from traditional learning to online learning), it is of special importance to manage one’s internal and external resources for learning.

Resource management strategies are strategies that aim to manage and control one’s learning environment (Pintrich, 1999; Vrugt and Oort, 2008). These include regulation of internal resources (effort, motivation) as well as external resources (study environment, time management, and help-seeking). Significant relations with academic achievement (Vrugt and Oort, 2008; Waldeyer et al., 2019), especially in online learning settings (Tsai et al., 2013; Broadbent and Poon, 2015), demonstrate the importance of resource management strategies in higher education. In the following, we focus especially on external resource management strategies.

When learning online and at a distance, students do not have access to a structured learning environment like classrooms, libraries, and learning and computer labs. They need to regulate their physical learning environment; that is, they need to decide where to study – e.g., in which room and ideally with no or limited possibilities for distraction (Lynch and Dembo, 2004). If lectures, rather than being physical live sessions, are recorded and if communication takes place online (either synchronous or asynchronous), timetables will need to be rescheduled and students will need to keep track of their time management (Song et al., 2004; Mahasneh et al., 2012; Kim et al., 2019). If physical isolation leads to low social presence, interactions with peers and lecturers might be hindered and students might remain invisible (Bedenlier et al., 2020). In addition, when students need help, they are required to develop other strategies of help-seeking than they typically would in regular physical interaction. However, online-based help-seeking might also have advantages that lead to more frequent use of help-seeking strategies (Kitsantas and Chow, 2007; Hao et al., 2016). For example, asynchronous communication allows for the posing of questions at any time – with the caveat that answers to those questions might not be immediately provided. With regard to seeking help from persons of higher status (teachers), the lack of social status cues might serve to facilitate help-seeking behavior (Mahasneh et al., 2012). Current research found low levels of interaction, while teacher-student interaction happened more often than student-student interaction (Hamdan et al., 2021).

To gain insights into self-regulated learning and resource management strategies especially in online higher education, existing questionnaire instruments are contextualized to the online or blended learning environment (Barnard et al., 2009; Jansen et al., 2017). Research before emergency remote teaching and learning focused on (the development of) both self-regulated learning within specific online learning environments like massive open online courses or blended learning scenarios and self-regulated learning’s relationship with academic achievement (Tabuenca et al., 2015; Kizilcec et al., 2017; Garcia et al., 2018; Jivet et al., 2020). In addition, the use of self-regulated learning strategies between different types of online education like fully online vs. blended or traditional courses is compared (Broadbent, 2017). Results of those studies, however, might not be transferrable to the situation of emergency remote teaching. Before the pandemic, students voluntarily self-selected online or distance education. Usually, students studying in online (distance) courses resemble a different student population than traditional on-site students. Those differences are contingent upon on age, vocational education, work situation, or family situation (Yukselturk and Top, 2012; Broadbent, 2017). That is, it remains unclear how traditional students would cope with the shift from traditional on-site courses to (asynchronous) online courses.

Still, the results of those studies provide interesting insights into self-regulated learning in online education. First, keeping in mind the limitations regarding student characteristics, it seems that strategy application differs between different delivery formats of education. For example, Broadbent (2017) found that students participating in online settings used time management strategies more often compared to students learning in blended learning settings. Regarding help-seeking strategies, the literature provides heterogeneous findings: in the studies by Shea and Bidjerano (2012) and Broadbent (2017), help-seeking strategies were more often implemented in blended compared to online learning. This contrasts with findings by Kitsantas and Chow (2007), both of whom investigated several perspectives of intended help-seeking behavior. It is likely that social presence coupled with the modality of the courses are moderators of help-seeking behavior (Shea and Bidjerano, 2012; Molinillo et al., 2018). According to several empirical studies, there exists a strong connection between self-regulated learning and students’ digital readiness. Anthonysamy et al. (2020b) demonstrated a significant and positive link between students’ cognitive, technical, and socio-emotional abilities in order to participate in online learning and students’ use of resource management strategies. In line with these findings, Greene et al. (2018) found evidence that self-regulated learning strategies play a major role in developing such skills for online learning. Likewise, Kiliç-Çakmak (2010) showed that the use of internal resource management strategies predicted students’ abilities to assess and communicate information. However, the study lacks information on external resource management strategies. To sum up, existing research focuses mostly on self-regulated learning strategies as prerequisite for students’ ability to develop online learning skills (Hung et al., 2010). In contrast to that focus, Muthupoltotage and Gardner (2018) investigated the interrelationship between the aforementioned skills and self-regulated learning strategies. They found empirical evidence that students’ technical and operational skills to participate in online learning predicted their use of strategies to structure their learning environment. In addition, students’ abilities to search and communicate information significantly predicted their use of help seeking strategies. However, the cited studies are methodically limited to cross-sectional surveys with one occasion of measurement and used instruments like the Motivated Strategies for Learning Questionnaire (MSLQ) that are not specifically designed to assess strategies in online learning settings.

Considering students’ experiences with online courses (number of online courses taken), higher education students in the study by Bruso et al. (2020) did not differ in their use of online self-regulated learning strategies (including resource management strategies). Similarly, a study with freshmen students in their first online term indicates that they did not improve in their use of self-regulated resource management strategies within one study term (Barnard-Brak et al., 2010). These non-significant pre-post comparisons regarding resource management strategies indicate that online courses do not necessarily foster self-regulated learning. In detail, students did not change their strategy use regarding environment structuring, time management, or help-seeking. Again, students in that study were older students who actively self-selected the online learning course and were aware of the required autonomy within the course. In addition, the offered course format was exclusively asynchronous, administered *via* an online learning course management and delivery system. Hence, it remains unclear how those results transfer to students’ resource management within a non-voluntary situation of studying remotely.

### Aims and Research Questions

The current study took place during an exceptional situation of emergency remote teaching and learning. Students neither actively decided nor were they prepared for a digital semester. Hence, the current study investigated students’ readiness for digital learning, students’ self-regulated learning, and the relationship between the two. In detail, we posed the following research questions:

First, to gain insights into higher education student readiness for online learning, we investigated students’ equipment, prior experiences with online learning, and their information-sharing behavior (ISB).

Q1: How ready are higher education students for online learning?

Second, we investigated students’ implementation of external resource management strategies during the term and how this related to students’ intentions before they experienced emergency remote teaching.

Q2a: To what extent do students plan and implement different external resource management strategies when experiencing emergency remote teaching?
Q2b: Does students’ use of external resource management strategies during the term differ from their intended use of external resource management strategies before entering emergency remote teaching?

Third, we examined whether students’ readiness for online learning is relevant to their use of resource management strategies during the term in which they faced emergency remote teaching.

Q3: How do students’ availability of equipment, previous experience with online learning, and information sharing behavior influence their self-regulated learning within remote emergency teaching?

## Materials and Methods

### Participants

We recruited students from one large, full-scale German university. Considering only students who completed both the first and second measurement, the number of participants was *N* = 662. Their mean age at the first assessment was 22.9 years (*SD* = 4.7) across all semesters; 66.8% of participants were female students. Across all five faculties of the university, students participated voluntarily in the survey (Faculty of Humanities, Social Sciences, and Theology: *n* = 181; Faculty of Sciences: *n* = 93; Faculty of Business, Economics, and Law: *n* = 140; Faculty of Engineering: *n* = 140; Faculty of Medicine: *n* = 108). Similarly, students from different study programs participated in the online survey (bachelor: *n* = 247; master: *n* = 154; state exam: *n* = 235; doctoral degree: *n* = 8; and others: *n* = 11). About 5% of the students lived with children with on-site childcare. The distribution of students across faculties, study programs, and students with/without children is in accordance with the university’s student population.

### Procedure

**Figure 1:**
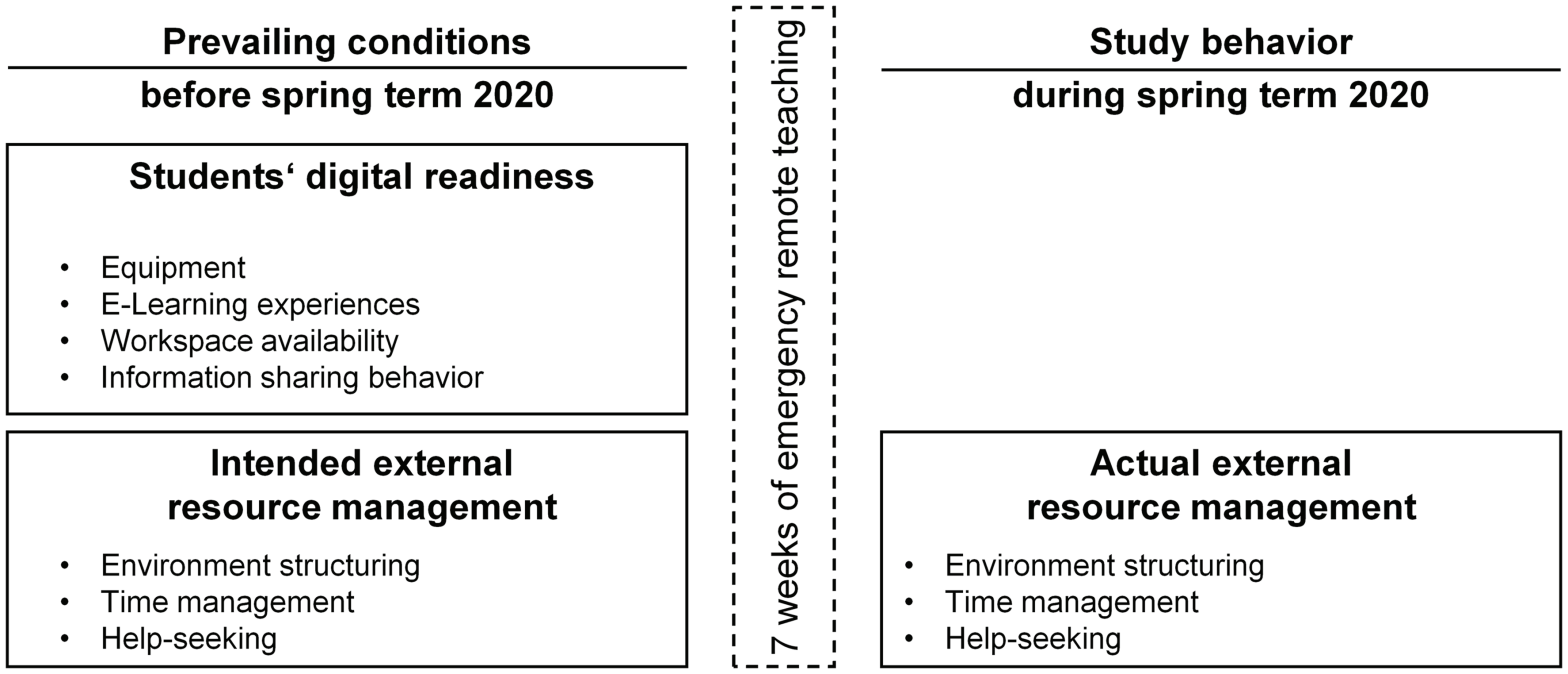
Assessed constructs and variables at both measurement time points.

This paper reports on the results of a longitudinal, pre-registered study^1^ during the spring term 2020 in Germany. University students participated in an online survey with three measurements. To answer the research question, the current study focuses on the first two of three measurements, namely the measurements in April 2020 and in June 2020 (the middle of the term), directly before the spring term 2020 had started and when students had already completed 7 weeks of online courses, respectively (see Figure 1). Students were informed that each online survey would take approximately 20 min and that the survey concerns student learning in the sudden online term. The online survey was administered in the German language and was rolled out *via* the platform Unipark Questback EFS.^2^

We protected participants’ privacy in accordance with the institutional commissioner for data protection. Students were not disadvantaged because of non-participation. At the beginning of the questionnaire, the participants gave their informed consent to participate in the study. All data were in pseudonymous form and students yielded an individual password for data matching purposes.

### Instruments

The online survey comprised socio-economic information and several standardized scales. First, students provided information on their individual backgrounds and on their current choice of study. We assessed age, gender, children, and study-related factors (current semester, faculty of the study program enrolled in, and intended degree). Next, students answered questionnaire scales to assess digital readiness as prerequisites and management of resources as outcomes of their learning. Students answered questions about their technical equipment, previous experiences with online learning tools, availability of a quiet workspace, and about their information sharing behavior. Finally, we assessed students’ use of external resource management strategies *via* three subscales (environment structuring, time management, and help-seeking).

#### Students’ Digital Readiness

Students’ workspace availability, equipment, previous experiences with online learning, and information sharing behavior were indicators of students’ readiness to engage in online learning. These variables represented the prevailing conditions with which students started the emergency remote term and include both external (workspace and equipment) and personal factors (experiences and information sharing behavior).

##### Workspace Availability

We asked students about the availability of a workspace that offered the possibility to study without disruption. The item was dichotomous (0 “no workspace available,” 1 “available workspace”).

##### Equipment

Regarding students’ equipment, we assessed whether they had access to certain devices or not, namely desktop PC, notebook, tablet, smartphone, scanner, printer, and internet access. The variable equipment represents the sum score of available devices and ranged from 0 to 7.

##### E-learning Experiences

We assessed students’ previous experiences with online learning in a similar manner. Students rated whether they had been working with one of the following tools of online learning before the spring term of 2020: downloadable scripts, recorded lectures, livestreams of lectures, use of digital live voting or live feedback, online communication and online teamwork, electronic exams, and online self-testing. The variable E-learning reflects the sum score of online learning features, students had experienced before entering the remote emergency term. The score ranged from 0 “no experiences” to 9 “experiences with all tools.”

##### Information-Sharing Behavior

As an additional indicator for students’ digital readiness, we focused on the scale for ISB from the digital readiness for academic engagement questionnaire (Hong and Kim, 2018). The scale comprised four items and was internally consistent with Cronbach’s *α*_1_ = 0.82. An example item was: “I can interact with classmates using real-time communication tools, for example, video conferencing tools or messengers.” Students answered the items on a six-point Likert scale, ranging from 1 “not true at all” to 6 “absolutely true.”

#### Online Self-Regulated Learning

The online self-regulated learning questionnaire (OSLQ; Barnard et al., 2009) measures self-regulated learning in the online learning environment as active and volitional behavior for learning successfully. We focused on three subscales addressing strategies used for external resource management according to the theoretical framework by Winne and Hadwin (1998), namely environment structuring, time management^3^, and help-seeking. In contrast to other taxonomies of self-regulated learning strategies (e.g., Waldeyer et al., 2019), we included time management as an external resource rather than an internal resource because. Students answered all items of the three subscales: environment structuring, time management, and help-seeking, at both measurements; that is, before as well as during the term. The only difference was how we introduced the items. Before the courses had started in April 2020, we asked students to think about how they plan to learn in online environments in the upcoming term. During the term, students reported on their actual learning behavior. All items had to be answered on a six-point Likert scale, ranging from 1 “not true at all” to 6 “absolutely true.”

First, the *environment structuring* scale focused on strategies regarding the organization and choice of students’ learning environment. An example for an item: “I know where I can study most efficiently for online courses.” The internal consistency of the four-item scale was satisfying, Cronbach’s *α*_1_ = 0.73, *α*_2_ = 0.82.

Second, the *time management* scale focused on students’ strategies for organizing their schedules and managing their times of study considering asynchronous and synchronous online courses. An item that focused on the challenges of synchronous and asynchronous online courses included: “Although we don’t have to attend daily classes, I still try to distribute my studying time evenly across days.” The internal consistency of the four-item scale was acceptable but low, Cronbach’s *α*_1_ = 0.60, *α*_2_ = 0.66.

Third, the *help-seeking* scale focused on students’ tendency to ask peers and instructors for help regarding the content of their online courses. We used the modified help-seeking scale from factor analysis of Jansen et al. (2017). Items of both scales inquired about student communication when faced with problems during study periods. A sample item was: “I share my problems with my classmates in this course online so we know what we are struggling with and how to solve our problems.” This scale consisted of five items and its internal consistency was satisfactory, Cronbach’s *α*_1_ = 0.74, *α*_2_ = 0.76.

### Data Analysis

Descriptive statistics on students’ equipment, their previous experiences with online learning tools, availability of a quiet workspace as well as their score on the scale of information sharing behavior answered the research question concerning students’ overall readiness to engage in online learning (Q1). We analyzed the descriptive statistics of the three external resource management strategies on both measurement occasions to answer research question Q2a. To answer the research question Q2b, we conducted a repeated measures multivariate ANOVA (MANOVA) showing differences between students’ intended and actual use of resource management strategies during the emergency remote term. The analysis consisted of one independent factor time with two distinctions (before and during emergency remote teaching) and three dependent variables (environment structuring, time management, and help-seeking). Finally, we calculated separate regression analyses to investigate the prediction of students’ use of resource management strategies during the term based on their prevailing conditions (Q3). We regressed actual study behavior (i.e., use of resource management strategies in the middle of the study term) on students’ workspace availability, equipment, previous e-learning experiences, and information sharing behavior. Additionally, we checked for effects by students’ gender and age.

## Results

### Students’ Readiness for Emergency Remote Teaching

Table 1 shows all mean scores concerning indicators of students’ readiness to engage in online learning. On average, students had access to many devices (i.e., six out of seven). Only five students either had no internet access or had no access to a desktop PC, notebook, or tablet to participate in online courses. However, all five students owned a smartphone. Students had experienced approximately half of the online learning features provided by the university. In addition, the majority of students had access to a quiet workspace. In consideration of the information sharing behavior, students rated their ability to communicate online as rather high.

**Table 1:** Descriptive statistics for all aspects of students’ digital readiness.

Digital readiness	*M* (*SD*)
External	
Workspace	0.93 (0.26)
Equipment	6.09 (1.17)
Personal	
E-Learning	4.88 (1.97)
Information Sharing Behavior	5.03 (0.88)

Table 2 shows all correlations between indicators of students’ digital readiness and their use of resource management strategies during the term. External indicators significantly correlated with students’ use of environment structuring strategies. Considering the internal indicators of students’ digital readiness, only their information sharing behavior significantly correlated with environment structuring and help-seeking. The correlations between students’ intended and actual use of external resource management strategies were significant and of moderate sizes, *r* = 0.20–0.64, *p* < 0.01.

**Table 2:** Correlations between indicators of students’ digital readiness and their use of resource management strategies during the term.

	Workspace	Equipment	E-learning	ISB	Environment structuring	Time management	Help-seeking
Workspace	1						
Equipment	0.21^**^	1					
E-learning	0.09^*^	0.11^**^	1				
ISB	0.04	0.16^**^	0.15^**^	1			
Environment structuring	0.18^**^	0.14^**^	0.07	0.08^*^	1		
Time management	0.02	0.01	0.04	−0.03	0.49^**^	1	
Help-seeking	−0.01	−0.07	0.07	0.13^**^	0.26^**^	0.26^**^	1

ISB, information sharing behavior.

* *p* < 0.05;

** *p* < 0.01.

### Intended vs. Implemented Resource Management Strategies

Table 3 presents the descriptive statistics concerning resource management. At the first measurement, that is, before online lectures had started, students intended to use all three types of strategies to a moderate degree. Most often, they planned to implement strategies to structure their learning environment. Moreover, students structured their environment most frequently and were least likely to seek help during the term.

**Table 3:** Descriptive statistics and results of a repeated measures multivariate ANOVA (MANOVA) comparing intended and actual use of external resource management strategies.

	*M*_Before_ (*SD*)	*M*_During_ (*SD*)	*F*	*p*	η_p_*^2^*
Environment structuring	4.50 (0.83)	4.50 (0.89)	0.02	0.902	0.00
Time management	4.18 (0.89)	4.10 (1.06)	3.91	0.049	0.01
Help-seeking	3.88 (0.90)	3.52 (1.00)	142.08	0.000	0.18

The repeated measures MANOVA indicated that students showed lower use of online SRL during the term than they intended to use before entering emergency remote teaching. This difference was statistically significant, *F*(3,657) = 36.05, *p* < 0.001, η_p_^2^ = 0.14. Table 3 indicates that the use of environment structuring strategies did not significantly differ when comparing intended strategy use before the emergency remote teaching and actual study behavior. However, the intended vs. actual use of time management strategies showed a small but significant difference. The difference between intended and actual help-seeking was significant and yielded a large effect. That is, students made less frequent use of help-seeking strategies than they planned to.

### Influence of Students’ Digital Readiness on Resource Management

Separate regression analyses to analyze potential predictors for the actual use of each resource management strategy showed varying results (see Table 4). Overall, gender was a significant predictor for each external resource management strategy. This indicates that women structured their learning environment, managed their time, and asked for help more frequently than males in the current sample. Age, in contrast, was not significantly related to any of the three strategies. The predictors significantly explained 7 % of the variance in the use of environment structuring strategies. The standardized betas showed that the availability of a quiet workspace was the strongest significant predictor followed by students’ equipment. Having access to a higher number of electronic devices and being able to use a quiet workspace led to a more frequent use of strategies to organize the learning environment. Regarding time management, no aspect of students’ digital readiness predicted any variance in students’ organization of study time significantly. Finally, on students’ help-seeking during the term, the predictors significantly explained 2 % of the variance in the use of this strategy. Students’ ability to communicate online was the strongest significant predictor followed by students’ equipment. While a higher score on the scale of information sharing behavior led to a higher number of strategies used during the term, having a lower number of electronic devices resulted in more help-seeking strategies.

**Table 4:** Separate regression analyses to predict strategy use during the term based on students’ digital readiness.

Variable	*B*	95% CI for *B*		*SE B*	*β*	*R*^2^
		LL	UL			
**Environment structuring**						0.07^***^
Constant	2.35^***^	1.60	3.11	0.38		
Workspace	0.68^***^	0.40	0.95	0.14	0.19^***^	
Equipment	0.08^*^	0.02	0.14	0.03	0.10^*^	
E–learning	0.01	−0.02	0.05	0.02	0.03	
ISB	0.07	−0.01	0.16	0.04	0.07	
Age	0.01	−0.01	0.02	0.01	0.03	
Gender	0.30^***^	0.15	0.46	0.08	0.16^***^	
**Time management**						0.04^***^
Constant	2.99^***^	2.10	3.88	0.45		
Workspace	0.10	−0.23	0.42	0.17	0.02	
Equipment	0.02	−0.06	0.09	0.04	0.02	
E–learning	0.01	−0.03	0.07	0.02	0.02	
ISB	−0.02	−0.11	0.08	0.05	−0.01	
Age	0.01	−0.01	0.02	0.01	0.03	
Gender	0.47^***^	0.29	0.65	0.09	0.21^***^	
**Help-seeking**						0.03^**^
Constant	3.14^***^	2.29	3.99	0.43		
Workspace	−0.02	−0.33	0.29	0.16	−0.01	
Equipment	−0.08^*^	−0.15	−0.05	0.04	−0.09^*^	
E–learning	0.03	−0.01	0.07	0.02	0.06	
ISB	0.12^**^	0.03	0.22	0.05	0.11^**^	
Age	−0.02	−0.03	0.00	0.01	−0.08	
Gender	0.24^**^	0.07	0.41	0.09	0.11^**^	

ISB, information sharing behavior. Gender 1 = men, 2 = women.

* *p* < 0.05;

** *p* < 0.01;

*** *p* < 0.001.

## Discussion

In the current study, we investigated students’ readiness when facing sudden online learning and their self-regulated use of resources during the remote emergency term in 2020. We assessed student strategy application twice – before online courses had started and in the middle of the term when students already had experienced online teaching and learning. The study revealed that students faced multiple challenges concerning the management of their resources, and they intended to use more regulating activities than were actually employed during the term. In addition, students’ digital readiness significantly predicted students’ implementation of resource management strategies. In the following, we discuss the results regarding each research question separately.

### Summary and Discussion of Results

To answer research question Q1, the present study addressed students’ readiness to participate in online learning, which arose through emergency remote teaching in the upcoming term. Students reported adequate access to external indicators of digital readiness. The majority of students had access to a quiet workplace to study for their courses and they reported a relatively high number of available electronic devices. Almost every student owned adequate electronic devices to access online learning platforms and to participate in asynchronous and synchronous online courses. However, the current study used an online questionnaire, which limited study participation to students who had access to the internet, at least while answering the two surveys. Students’ devices should have enabled them to stream videotaped lectures, discuss topics with their fellow students online, follow up on online courses, or do their coursework. However, the two personal indicators of student digital readiness varied. Students’ experiences with online learning features before the spring term of 2020 were limited. On average, students had used slightly more than half of the features listed in the survey. Furthermore, and in accordance with Kim et al. (2019), students were confident that they could communicate online. Lee and Tsai (2011) also demonstrated a rather high-perceived capability among students to communicate online with their peers. Accordingly, German higher education students are ready to enter the remote emergency term regarding their equipment, workspace, and their ability to communicate with their peers and instructors. This is particularly true when considering the short preparation time before the emergency remote teaching.

To investigate the second research question (Q2a, Q2b), we assessed students’ management of external resources before entering the emergency remote term as well as within the term. In summary, repeated measures MANOVA demonstrated that students had trouble managing their resources as they intended to for the situation at hand. Two assumptions might explain the results: Students either did not see the relevance of using the strategies or were actually not able to use them. Regarding the first assumption, students might expected the emergency remote teaching to take more time and cause more problems than was actually the case. Using fewer resource management strategies could be sufficient for successful participation in new courses if instructors implemented them with care. Regarding the second assumption, students might know which strategies they wanted to use but lacked the ability to apply these strategies in a novel online learning environment (production deficit, Veenman, 2007). In addition, it is conceivable that emergency remote teaching required other strategies with which students were not familiar (availability deficit, Veenman, 2007). Interestingly, all mean scores of the three implemented scales of resource strategies were lower than students’ self-reported scores of information sharing behavior, indicating that the results regarding resource management strategies did not occur because of general answer biases.

It was mostly the case that students intended to implement strategies that structure the learning environment and did in actuality, implemented said strategies. Current research demonstrates significant relations between environment structuring among other resource management activities and academic achievement in offline and online learning (Vrugt and Oort, 2008; Tsai et al., 2013; Broadbent and Poon, 2015; Waldeyer et al., 2019). Students might recognize the importance of this strategy, and therefore intended to use and did, in fact, use it most frequently. Furthermore, a well-structured learning environment is a basic condition for studying that is (ideally) available to students every day.

Although time management strategies in our study were used less often than students’ intended, promising results from a study by Zhang et al. (2021) indicate that students still managed to complete their assignments in the first remote emergency term. Additionally, efficient time management is essential to handle procrastination and leads to higher academic achievement (Wolters et al., 2017; Wolters and Brady, 2020). Hong et al. (2021) demonstrated that procrastination predicts students’ application of resource management strategies during the COVID-19 pandemic. Students who engaged in more procrastination notably used fewer time management strategies and rated their learning effectiveness significantly lower.

Regarding help-seeking strategies, the results are comparable to those by Hamdan et al. (2021) who found that interaction between peers especially, was rather low in higher education during the COVID-19 pandemic. However, help-seeking is referring to having a problem (e.g., difficulties in understanding) while interaction is a more broad construct not only encompassing help-seeking but also personal interaction concerning other aims. On the one hand, low help-seeking behavior might imply that students do not need to seek help (Stahl and Bromme, 2009), probably because of low task difficulty (Hao et al., 2016). On the other hand, because of a lack of opportunities for on-site meetings, students might not have developed adequate strategies to seek help during online and distance learning.

Regarding research question Q3, we demonstrated that indicators of students’ readiness for online learning significantly predicted their resource management during the emergency remote term. First, students’ availability of a quiet workspace predicted a more frequent application of strategies to structure their learning environment. Additionally, students who had more electronic devices available used more strategies to structure their learning environment but implemented less help-seeking strategies. It is possible that those students already had a permanent place for their devices and constantly structured their learning environment in a way that separated their private and academic use of said devices. However, a higher number of accessible devices resulted in significantly lower use of strategies of help-seeking during the term. It is likely that students with more electronic devices available used their devices more frequently and were able to take advantage of every opportunity to search for information on academic courses (e.g., website and chatrooms). Therefore, they might not have needed additional content related assistance from peers or instructors. Students who scored higher regarding their ability to communicate online used more help-seeking strategies to get in contact with their fellow students and instructors during the term. This underlines that students’ information sharing behavior in online environments reflects a basic skill that students require to engage in help-seeking (Hong and Kim, 2018; Muthupoltotage and Gardner, 2018).

Students’ previous experiences with online learning did not significantly predict the emergence or application of any of the resource management strategies. This is in line with findings of Bruso et al. (2020) indicating that differences in students’ previous experiences with online learning do not necessarily correlate with their use of self-regulated learning. Finally, none of the aspects of students’ digital readiness predicted their use of time management strategies significantly. Wolters et al. (2017) demonstrated that students’ time management correlates with their procrastination. Students who reported a low preference for the organization of their study time as well as for their goal-setting showed a higher tendency to procrastinate. According to Wolters and Brady (2020), students’ time management is closely related to each phase of student learning and significantly predicts academic achievement. Thus, there are many possibilities to promote students’ time management. Students’ digital readiness does not seem to be an effective starting point.

Additionally, the regression analysis showed that women in our sample used more strategies in all three subscales of the assessed external resource management. This is in line with findings by Bidjerano (2005) who showed that female undergraduates reported to use significantly more time management and environment-structuring strategies than their male counterparts did. In addition, our study also revealed gender differences with regard to help seeking strategies (in favor of female students). Our results are first indicators that gender differences are also evident in the context of emergency remote teaching and should be considered by future research on learning strategies in online learning contexts.”

### Limitations and Prospects for Future Research

The aim of the current study is to provide early insight into students’ (intended) behavior before and within the first online term during the COVID-19 pandemic. Results clearly indicated that there is room for improvement regarding students’ use of resource management strategies, especially when it comes to their use during the term. In light of previous research that convincingly demonstrated the importance of resource management strategies for academic performance (Tsai et al., 2013; Broadbent and Poon, 2015), and assuming that this applies to the situation of emergency remote teaching as well, the current study results underline the necessity for training student implementation of resource management strategies. A limitation of the current research, however, is that it does not provide empirical evidence regarding the relationship of the application of resource management strategies and academic achievement in the situation of emergency remote teaching. Taking into account that women used significantly more strategies than men did, it would be of special interest if this more frequent use of external resource management strategies leads to higher academic achievement. However, the current study used a broad sampling strategy encompassing students of various disciplines and study programs. Consequently, students’ use of resource management strategies was not linked to specific courses or course performance as is the case, for example, in studies with more process-based assessments (Loeffler et al., 2019). Additionally, we assessed learning strategies *via* self-report questionnaires that only contain global information about the use of the listed strategies and that are limited concerning students’ true use of these strategies (Rovers et al., 2019). Still, in order to provide an assessment as situation-specific as possible within a large student sample, we used well-established questionnaire scales that refer to the specific situation of online learning in higher education. In addition, we explicitly asked students to provide their answers with regard to their actual use of strategies in the instruction. Assuming that emergency remote teaching leads to a boost of online higher education or at least to more hybrid formats in the near future, current research should inherit more situation-specific perspectives to disentangle which strategies are most important and consequently, would need support. Specifically, despite the current study’s use of established scales in the context of online learning, the results are limited to self-report of strategy use, and the scale time management showed rather low internal consistency for both measurement occasions. In addition, we assessed only a subset of the self-regulated learning strategies used by students when experiencing emergency remote teaching. Nevertheless, we claim the strategies in our study as essential regarding external resource management.

The relatively low mean scores for the use of external resource strategies points to a need to support students’ self-regulated resource management in online higher education (Karabenick, 2011; Wong et al., 2019). In general, three approaches seem conceivable to promote students’ resource management. First, the regression analyses of this study revealed significant predictions of indicators of students’ digital readiness on their actual study behavior. Therefore, an implication would be to promote digital readiness among students to strengthen the use of resource management strategies. However, our results indicate only small significant effects and low proportions of explained variance in the use of resource management strategies. For this reason, the disadvantage could be marginal as long as students fulfill basic conditions (e.g., one electronic device and internet access). Second and third, the training literature on self-regulated learning suggests (a) direct and (b) indirect approaches that have even been shown to have transfer effects for cognitive and metacognitive strategies (Schuster et al., 2018, 2020; Dignath and Veenman, 2020). For example, the university where we conducted this research offers self-regulated learning courses for their students. In these courses, students learn about the conditions and processes of selected learning strategies. van der Beek et al. (2019) demonstrated that such courses promote self-regulated learning whether they take place in-person or online. Restructuring online learning that facilitates students’ use of resource management strategies, on the other hand, is one approach to indirect training. A low-threshold and suitable tool with which to support students as they reflect on their learning situation and learning progress includes e-portfolios (Gläser-Zikuda et al., 2011; Händel et al., 2020b). Students reflect on their learning behavior and in doing so, have the possibility to be made aware of their strengths and difficulties. This might help students to regulate their resources. If students are having difficulty asking others for help, prompts might encourage them to ask questions, which could in turn, lower the threat presented by the need to ask for help (Schworm and Gruber, 2012). The establishment of smaller learning groups could very well be an opportunity to encourage students’ to interact with one another and/or seek help (e.g., *via* breakout sessions on videoconferencing platforms). Oviedo and Fox Tree’s (2021) current study suggests that communication *via* video-chat improves student perception of conversations on coursework and of their efficiency when working together.

### Conclusion

Overall, this study offers early insights into how students coped with the situation of emergency remote teaching; that is, how they regulated their resources. It revealed that students were digitally ready for online learning but were not able to apply as many resource regulation strategies as initially intended. In light of the importance of the use of strategies for academic achievement, we discussed several approaches with which to assist students in their regulation of learning resources. We think that low-threshold measures (e.g., small group sizes, prompts, etc.) along with a basic digital readiness are simple, efficient, and direct implementations in online courses. Still, online learning settings significantly differ from regular higher education situations due to physical distancing and fundamentally different forms of communication. Hence, training methods exclusively developed for distance education might be necessary and helpful (van der Beek et al., 2019).

## Data Availability Statement

The raw data supporting the conclusions of this article will be made available by the authors, without undue reservation.

## Ethics Statement

Ethical review and approval was not required for the study on human participants in accordance with the local legislation and institutional requirements. The patients/participants provided their written informed consent to participate in this study.

## Author Contributions

MH, AZ, MG-Z, BK, RK, and SB designed the study. MH and BK carried out the data collection. NN and MH performed the data analyses and were major contributors in writing the manuscript. All authors contributed to the article and approved the submitted version.

### Conflict of Interest

The authors declare that the research was conducted in the absence of any commercial or financial relationships that could be construed as a potential conflict of interest.

## Footnotes

^1^https://osf.io/68aus/

^2^unipark.com

^3^In emergency remote teaching external factors (e.g., deadlines and limited time on task) predetermine the extent to which students have to regulate their study time (Wolters and Brady, 2020). Therefore, in accordance with the model by Winne and Hadwin (1998), we assign time management to the external resource management strategies even though other taxonomies of self-regulated learning strategies consider time management as internal resource (e.g., Waldeyer et al., 2019).

## References

[ref1] Anthonysamy L. Koo A.-C. Hew S.-H. (2020a). Self-regulated learning strategies and non-academic outcomes in higher education blended learning environments: a one decade review. Educ. Inf. Technol. 25, 3677–3704. 10.1007/s10639-020-10134-2

[ref2] Anthonysamy L. Koo A.-C. Hew S.-H. (2020b). Self-regulated learning strategies in higher education: fostering digital literacy for sustainable lifelong learning. Educ. Inf. Technol. 25, 2393–2414. 10.1007/s10639-020-10201-8

[ref3] Barnard L. Lan W. Y. To Y. M. Paton V. O. Lai S.-L. (2009). Measuring self-regulation in online and blended learning environments. Internet High. Educ. 12, 1–6. 10.1016/j.iheduc.2008.10.005

[ref4] Barnard-Brak L. Paton V. O. Lan W. Y. (2010). Self-regulation across time of first-generation online learners. Res. Learn. Technol. 18, 61–70. 10.1080/09687761003657572

[ref5] Beaunoyer E. Dupere S. Guitton M. J. (2020). COVID-19 and digital inequalities: reciprocal impacts and mitigation strategies. Comput. Hum. Behav. 111:106424. 10.1016/j.chb.2020.106424, PMID: 32398890 PMC7213963

[ref6] Bedenlier S. Wunder I. Gläser-Zikuda M. Kammerl R. Kopp B. Ziegler A.. (2020). “Generation invisible“. Higher education students’ (non)use of webcams in synchronous online learning. PsyArXiv [Preprint]. 10.31234/osf.io/7brp6

[ref7] Bidjerano T. (2005). “Gender differences in self-regulated learning,” in *Annual Meeting of the Northeastern Educational Research Association*; October 19–21, 2005; New York, United States: Kerhonkson.

[ref8] Bol L. Garner J. K. (2011). Challenges in supporting self-regulation in distance education environments. J. Comput. High. Educ. 23, 104–123. 10.1007/s12528-011-9046-7

[ref9] Bozkurt A. Jung I. Xiao J. Vladimirschi V. Schuwer R. Egorov G.. (2020). A global outlook to the interruption of education due to COVID-19 pandemic: navigating in a time of uncertainty and crisis. Asian J. Distance Educ. 15, 1–126. 10.5281/zenodo.3878572

[ref10] Broadbent J. (2017). Comparing online and blended learner’s self-regulated learning strategies and academic performance. Internet High. Educ. 33, 24–32. 10.1016/j.iheduc.2017.01.004

[ref11] Broadbent J. Poon W. L. (2015). Self-regulated learning strategies & academic achievement in online higher education learning environments: a systematic review. Internet High. Educ. 27, 1–13. 10.1016/j.iheduc.2015.04.007

[ref12] Bruso J. Stefaniak J. Bol L. (2020). An examination of personality traits as a predictor of the use of self-regulated learning strategies and considerations for online instruction. Educ. Technol. Res. Dev. 68, 2659–2683. 10.1007/s11423-020-09797-y

[ref13] Carter J. R. A. Rice M. Yang S. Jackson Haidee A. (2020). Self-regulated learning in online learning environments: strategies for remote learning. Inf. Learn. Sci. 121, 321–329. 10.1108/ILS-04-2020-0114

[ref14] Czerniewicz L. Agherdien N. Badenhorst J. Belluigi D. Chambers T. Chili M.. (2020). A wake-up call: equity, inequality and Covid-19 emergency remote teaching and learning. Postdigital Sci. Educ. 2, 946–967. 10.1007/s42438-020-00187-4 PMC7509221 40477091

[ref15] Dignath C. Veenman M. V. J. (2020). The role of direct strategy instruction and indirect activation of self-regulated learning—Evidence from classroom observation studies. Educ. Psychol. Rev. 33, 489–533. 10.1007/s10648-020-09534-0

[ref16] Dresel M. Schmitz B. Schober B. Spiel C. Ziegler A. Engelschalk T.. (2015). Competencies for successful self-regulated learning in higher education: structural model and indications drawn from expert interviews. Stud. High. Educ. 40, 454–470. 10.1080/03075079.2015.1004236

[ref17] European Council (2006). Recommendation of the European Parliament and the Council on key competencies for lifelong learning. Off. J. Eur. Union. 49, 10–18.

[ref18] Garcia R. Falkner K. Vivian R. (2018). Systematic literature review: self-regulated learning strategies using e-learning tools for computer science. Comput. Educ. 123, 150–163. 10.1016/j.compedu.2018.05.006

[ref19] Gläser-Zikuda M. Fendler J. Noack J. Ziegelbauer S. (2011). Fostering self-regulated learning with portfolios in schools and higher education. Orbis Scholae 5, 67–78. 10.14712/23363177.2018.101

[ref20] Greene J. A. Copeland D. Z. Deekens V. M. Yu S. B. (2018). Beyond knowledge: examining digital literacy’s role in the acquisition of understanding in science. Comput. Educ. 117, 141–159. 10.1016/j.compedu.2017.10.003

[ref21] Hamdan K. M. Al-Bashaireh A. M. Zahran Z. Al-Daghestani A. AL-Habashneh S. Shaheen A. M. (2021). University students’ interaction, Internet self-efficacy, self-regulation and satisfaction with online education during pandemic crises of COVID-19 (SARS-CoV-2). Int. J. Educ. Manag. 10.1108/IJEM-11-2020-0513 [Epub ahead of print]

[ref22] Händel M. Stephan M. Gläser-Zikuda M. Kopp B. Bedenlier S. Ziegler A. (2020a). Digital readiness and its effects on higher education students’ socio-emotional perceptions in the context of the COVID-19 pandemic. J. Res. Technol. Educ. 10.1080/15391523.2020.1846147

[ref23] Händel M. Wimmer B. Ziegler A. (2020b). E-portfolio use and its effects on exam performance—a field study. Stud. High. Educ. 45, 258–270. 10.1080/03075079.2018.1510388

[ref24] Hao Q. Wright E. Barnes B. Branch R. M. (2016). What are the most important predictors of computer science students’ online help-seeking behaviors? Comput. Hum. Behav. 62, 467–474. 10.1016/j.chb.2016.04.016

[ref25] Hodges C. B. (2005). Self-regulation in web-based courses: a review and the need for research. Q. Rev. Dist. Learn. 6, 375–383.

[ref26] Hodges C. B. Moore S. Lockee B. B. Trust T. Bond A. (2020). The difference between emergency remote teaching and online learning. Educause Review. Available at: https://er.educause.edu/articles/2020/3/the-difference-between-emergency-remote-teaching-and-online-learning (Accessed April 20, 2021).

[ref27] Hong A. J. Kim H. J. (2018). College students’ digital readiness for academic engagement (DRAE) scale: scale development and validation. Asia Pac. Educ. Res. 27, 303–312. 10.1007/s40299-018-0387-0

[ref28] Hong J.-C. Lee Y.-F. Ye J.-H. (2021). Procrastination predicts online self-regulated learning and online learning ineffectiveness during the coronavirus lockdown. Personal. Individ. Differ. 174:110673. 10.1016/j.paid.2021.110673, PMID: 33551531 PMC7846229

[ref29] Hung M.-L. Chou C. Chen C.-H. Own Z.-Y. (2010). Learner readiness for online learning: Scale development and student perceptions. Comput. Educ. 55, 1080–1090. 10.1016/j.compedu.2010.05.004

[ref30] Jansen R. S. van Leeuwen A. Janssen J. Kester L. Kalz M. (2017). Validation of the self-regulated online learning questionnaire. J. Comput. High. Educ. 29, 6–27. 10.1007/s12528-016-9125-x

[ref31] Jivet I. Scheffel M. Schmitz M. Robbers S. Specht M. Drachsler H. (2020). From students with love: an empirical study on learner goals, self-regulated learning and sense-making of learning analytics in higher education. Internet High. Educ. 47:100758. 10.1016/j.iheduc.2020.100758

[ref32] Karabenick S. A. (2011). Classroom and technology-supported help seeking: the need for converging research paradigms. Learn. Instr. 21, 290–296. 10.1016/j.learninstruc.2010.07.007

[ref33] Kiliç-Çakmak E. (2010). Learning strategies and motivational factors predicting information literacy selfefficacy of e-learners. Australas. J. Educ. Technol. 26, 192–208. 10.14742/ajet.1090

[ref34] Kim H. J. Hong A. J. Song H.-D. (2019). The roles of academic engagement and digital readiness in students’ achievements in university e-learning environments. Int. J. Educ. Technol. High. Educ. 16:21. 10.1186/s41239-019-0152-3

[ref35] Kitsantas A. Chow A. (2007). College students’ perceived threat and preference for seeking help in traditional, distributed, and distance learning environments. Comput. Educ. 48, 383–395. 10.1016/j.compedu.2005.01.008

[ref36] Kizilcec R. F. Pérez-Sanagustín M. Maldonado J. J. (2017). Self-regulated learning strategies predict learner behavior and goal attainment in massive open online courses. Comput. Educ. 104, 18–33. 10.1016/j.compedu.2016.10.001

[ref37] Kocdar S. Karadeniz A. Bozkurt A. Buyuk K. (2018). Measuring self-regulation in self-paced open and distance learning environments. Int. Rev. Res. Open Dis. Learn. 19, 25–43. 10.19173/irrodl.v19i1.3255

[ref38] Küsel J. Martin F. Markic S. (2020). University students’ teadiness for using digital media and online learning—Comparison between Germany and the USA. Educ. Sci. 10:313. 10.3390/educsci10110313

[ref39] Lee S. W.-Y. Tsai C.-C. (2011). Students’ perceptions of collaboration, self-regulated learning, and information seeking in the context of Internet-based learning and traditional learning. Comput. Hum. Behav. 27, 905–914. 10.1016/j.chb.2010.11.016

[ref40] Loeffler S. N. Bohner A. Stumpp J. Limberger M. F. Gidion G. (2019). Investigating and fostering self-regulated learning in higher education using interactive ambulatory assessment. Learn. Individ. Differ. 71, 43–57. 10.1016/j.lindif.2019.03.006

[ref41] Lynch R. Dembo M. (2004). The relationship between self-regulation and online learning in a blended learning context. Int. Rev. Res. Open Dis. Learn. 5, 1–16. 10.19173/irrodl.v5i2.189

[ref42] Mahasneh R. A. Sowan A. K. Nassar Y. H. (2012). Academic help-seeking in online and face-to-face learning environments. E-Lear. Dig. Med. 9, 196–210. 10.2304/elea.2012.9.2.196

[ref43] Martin F. Sun T. Westine C. D. (2020). A systematic review of research on online teaching and learning from 2009 to 2018. Comput. Educ. 159:104009. 10.1016/j.compedu.2020.104009, PMID: 32921895 PMC7480742

[ref44] McBrien J. L. Cheng R. Jones P. (2009). Virtual spaces: employing a synchronous online classroom to facilitate student engagement in online learning. Int. Rev. Res. Open Dis. Learn. 10, 1–17. 10.19173/irrodl.v10i3.605

[ref45] Means B. Bakia M. Murphy R. (2014). Learning Online: What Research Tells us About Whether, When and How. London: Routledge.

[ref46] Means B. Neisler J. (2020). Unmasking Inequality: STEM Course Experience During the COVID-19 Pandemic. Digital Promise.

[ref47] Molinillo S. Aguilar-Illescas R. Anaya-Sánchez R. Vallespín-Arán M. (2018). Exploring the impacts of interactions, social presence and emotional engagement on active collaborative learning in a social web-based environment. Comput. Educ. 123, 41–52. 10.1016/j.compedu.2018.04.012

[ref48] Moore J. L. Dickson-Deane C. Galyen K. (2011). e-Learning, online learning, and distance learning environments: are they the same? Internet High. Educ. 14, 129–135. 10.1016/j.iheduc.2010.10.001

[ref49] Muthupoltotage U. P. Gardner L. (2018). “Analysing the relationships between digital literacy and self-regulated learning of undergraduates—a preliminary investigation,” in Advances in Information Systems Development: Methods, Tools and Management. eds. Paspallis N. Raspopoulos M. Barry C. Lang M. Linger H. Schneider C. (Cham: Springer).

[ref51] Oviedo V. Y. Fox Tree J. E. (2021). Meeting by text or video-chat: effects on confidence and performance. Comput. Human Behav. Rep. 3:100054. 10.1016/j.chbr.2021.100054

[ref52] Panadero E. (2017). A review of self-regulated learning: six models and four directions for research. Front. Psychol. 8:422. 10.3389/fpsyg.2017.00422, PMID: 28503157 PMC5408091

[ref53] Pintrich P. R. (1999). The role of motivation in promoting and sustaining self-regulated learning. Int. J. Educ. Res. 31, 459–470. 10.1016/S0883-0355(99)00015-4

[ref54] Pintrich P. R. Smith D. A. F. Garcia T. McKeachie W. J. (1991). A manual for the use of the Motivated Strategies for Learning Questionnaire (MSLQ). The University of Michigan.

[ref55] Rovers S. F. E. Clarebout G. Savelberg H. H. C. M. de Bruin A. B. H. van Merriënboer J. J. G. (2019). Granularity matters: comparing different ways of measuring self-regulated learning. Metacogn. Learn. 14, 1–19. 10.1007/s11409-019-09188-6

[ref56] Schuster C. Stebner F. Leutner D. Wirth J. (2020). Transfer of metacognitive skills in self-regulated learning: an experimental training study. Metacogn. Learn. 15, 455–477. 10.1007/s11409-020-09237-5

[ref57] Schuster C. Stebner F. Wirth J. Leutner D. (2018). Förderung des Transfers metakognitiver Lernstrategien durch direktes und indirektes Training [Fostering transfer of metacognitive learning strategies by direct and indirect training]. Unterrichtswissenschaft 46, 409–435. 10.1007/s42010-018-0028-6

[ref58] Schworm S. Gruber H. (2012). e-Learning in universities: supporting help-seeking processes by instructional prompts. Br. J. Educ. Technol. 43, 272–281. 10.1111/j.1467-8535.2011.01176.x

[ref59] Shea P. Bidjerano T. (2012). Learning presence as a moderator in the community of inquiry model. Comput. Educ. 59, 316–326. 10.1016/j.compedu.2012.01.011

[ref60] Song L. Singleton E. S. Hill J. R. Koh M. H. (2004). Improving online learning: student perceptions of useful and challenging characteristics. Internet High. Educ. 7, 59–70. 10.1016/j.iheduc.2003.11.003

[ref61] Stahl E. Bromme R. (2009). Not everybody needs help to seek help: surprising effects of metacognitive instructions to foster help-seeking in an online-learning environment. Comput. Educ. 53, 1020–1028. 10.1016/j.compedu.2008.10.004

[ref62] Tabuenca B. Kalz M. Drachsler H. Specht M. (2015). Time will tell: the role of mobile learning analytics in self-regulated learning. Comput. Educ. 89, 53–74. 10.1016/j.compedu.2015.08.004

[ref63] Tsai C.-W. Shen P.-D. Fan Y.-T. (2013). Research trends in self-regulated learning research in online learning environments: a review of studies published in selected journals from 2003 to 2012. Br. J. Educ. Technol. 44, E107–E110. 10.1111/bjet.12017

[ref64] van der Beek S. Bellhäuser H. Karlen Y. Hertel S. (2019). New ways in fostering self-regulated learning at university: how effective are web-based courses when compared to regular attendance-based courses? Z. Entwicklungspsychol. Padagog. Psychol. 34, 117–129. 10.1024/1010-0652/a000254

[ref65] Veenman M. V. J. (2007). The assessment and instruction of self-regulation in computer-based environments: a discussion. Metacogn. Learn. 2, 177–183. 10.1007/s11409-007-9017-6

[ref66] Vrugt A. Oort F. J. (2008). Metacognition, achievement goals, study strategies and academic achievement: pathways to achievement. Metacogn. Learn. 3, 123–146. 10.1007/s11409-008-9022-4

[ref67] Waldeyer J. Fleischer J. Wirth J. Leutner D. (2019). Validating the resource-management inventory (ReMI). Eur. J. Psychol. Assess. 36, 777–786. 10.1027/1015-5759/a000557

[ref68] Winne P. H. Hadwin A. F. (1998). “Studying as self-regulated engagement in learning,” in Metacognition in Educational Theory and Practice. eds. Hacker D. Dunlosky J. Graesser A. (Hillsdale, NJ: Erlbaum), 277–304.

[ref69] Wolters C. A. Brady A. C. (2020). College students’ time management: a self-regulated learning perspective. Educ. Psychol. Rev. 10.1007/s10648-020-09519-z

[ref70] Wolters C. A. Won S. Hussain M. (2017). Examining the relations of time management and procrastination within a model of self-regulated learning. Metacogn. Learn. 12, 381–399. 10.1007/s11409-017-9174-1

[ref71] Wong J. Baars M. Davis D. Van Der Zee T. Houben G.-J. Paas F. (2019). Supporting self-regulated learning in online learning environments and MOOCs: a systematic review. Int. J. Hum. Comput. Interact. 35, 356–373. 10.1080/10447318.2018.1543084

[ref72] Yukselturk E. Top E. (2012). Exploring the link among entry characteristics, participation behaviors and course outcomes of online learners: an examination of learner profile using cluster analysis. Br. J. Educ. Technol. 44, 716–728. 10.1111/j.1467-8535.2012.01339.x

[ref73] Zhang T. Taub M. Chen Z. (2021). Measuring the impact of COVID-19 induced campus closure on student self-regulated learning in physics online learning modules. arXiv [Preprint]. Available at: https://arxiv.org/abs/2101.05872v1 (Accessed April 25, 2021).

